# Characterization of moisture migration and diffusion in two types of tobacco biomass during the dehydration process by the TG-NMR analysis

**DOI:** 10.3389/fchem.2024.1367139

**Published:** 2024-03-12

**Authors:** Wenkui Zhu, Bo Zhou, Kun Duan, Duoduo Huang, Lifeng Han, Rongya Zhang, Wu Wen, Bing Wang, Bin Li

**Affiliations:** ^1^ Zhengzhou Tobacco Research Institute of CNTC, Zhengzhou, China; ^2^ China Tobacco Henan Industrial Co., Ltd, Zhengzhou, China; ^3^ China Tobacco Sichuan Industrial Co., Ltd, Chengdu, China

**Keywords:** drying, low field nuclear magnetic resonance, TG-NMR, adsorption energy, moisture statement

## Abstract

The tobacco waste generated from the tobacco agriculture and industry, including the discarded stem and leaf, often needs dehydration pretreatment before thermal conversion utilization. In order to study the water activity and migration of tobacco waste during the pretreatment process, TG-NMR (Thermogravimetric Nuclear Magnetic Resonance) was used to obtain the drying curves and LF-NMR (Low Field Nuclear Magnetic Resonance) T2 inversion spectrum at each stage of tobacco drying. Meanwhile, the variation pattern of pore distribution during the dehydration process of two types of tobacco waste has been obtained. Combined with the pore distribution changes, a possible spatial migration mode of water was proposed. The change of adsorption energy of water during tobacco drying was calculated, and verified the above hypothesis. This study results provide reference for the optimization of dehydration pretreatment process for different tobacco waste in order to reduce energy consumption during recycling of tobacco biomass.

## 1 Introduction

Tobacco is a major economic crop in the global market, with total production of 4.05 million tons in 2019 from major global tobacco producing areas (Glynn et al., 2021). At the same time, the waste of tobacco biomass is also very serious. Due to the particularity of tobacco crops, the utilization rate of tobacco plants is just 30%–35%, often only part of the leaves are used to process into tobacco products. While the stem and a small part of the leaves are discarded, as accounts for nearly 70% of the total biomass of tobacco plants. Therefore, the reuse of tobacco biomass has obvious economic value, such as energy utilization or converting tobacco waste into carbon-based materials (Charles et al., 2022; Zhao et al., 2022). For the recycling treatment process of tobacco biomass, the dehydration by different drying methods is often a necessary pre-treatment process, especially in thermal conversion and utilization (Amer et al., 2019; Gao et al., 2020). From the perspective of energy conservation, excessive water in tobacco biomass before thermal conversion processing will increase energy consumption. The moisture change of tobacco is the key point of attention. Understanding the water residence and migration patterns of tobacco materials is essential for reasonable optimization of tobacco biomass pretreatment process in order to reduce energy consumption during the waste recycling.

Tobacco is a naturally porous material and there are a large number of pores and duct structures in the tobacco matrix (Sakamoto and Nakanishi, 1993), in which moisture has various states of residence. In present, the TG analysis technique is commonly used to study the thermal weightlessness of tobacco during drying or pyrolysis, allowing to obtain the kinetic parameters, such as the apparent rate constant, diffusion coefficient and activation energy of the material (Yan et al., 2021). As a porous medium, the kinetic behavior of tobacco during dehydration is inextricably linked to the internal moisture migration and diffusion. However, TG analysis alone could not obtain the migration pattern and activity change of water during dehydration process. Low-field NMR is a fast and non-destructive measurement technique (Ezeanaka et al., 2019), which can reflect the moisture fugacity from microscopic point of view. Although it cannot achieve the function of high-resolution analysis and detection, it has the advantages of being inexpensive and does not require a highly non-uniform magnetic field and a large number of coil shielding, which has attracted wide attention from scholars in the field of food science research, and have been applied to the moisture content of agricultural products (Taghizadeh et al., 2009; Li et al., 2010), water distribution (Bertram et al., 2004; Han et al., 2004; Garnczarska et al., 2007; Hong et al., 2009), water activity (Pitombo and Lima, 2003a; Mao et al., 2020), and even used in agricultural production maturity (Charoensumran et al., 2021; Hu et al., 2020; Gao et al., 2020), drying moisture diffusion (Anisimov, 2021; Yao et al., 2022). Among them, by analyzing the difference of spin-spin relaxation time T2 of hydrogen protons of water molecules, for example, the shorter the spin relaxation time the weaker the activity of water and the longer the relaxation time the stronger the activity of water, it is easy to distinguish the water which is easy to flow loss and the water which is tightly bound by physical and chemical interactions (Sekiyama et al., 2012), so the spin-spin relaxation time T2 of hydrogen protons is used as an index for moisture content prediction and moisture activity evaluation (Mcconville and poo, 2001).

In this study, TG and Low-field NMR were coupled to study the changes of moisture activity and its migration behavior in two typical tobacco materials during the dehydration process. T21 peak evolution, which is the main peak in the LF-NMR T2 inversion spectrum of leaf and stem filaments, and their dehydration kinetics characteristics, were correlated and analyzed. The TG-NMR analysis results were further combined with pore distribution and water adsorption energy in tobacco materials to propose the law of moisture migration in tobacco leaf and stem filaments. This study provides a basic theoretical reference for moisture spatial migration in heat and humidity treatment of tobacco biomass.

## 2 Experiment

### 2.1 Sample preparation

**FIGURE 1:**
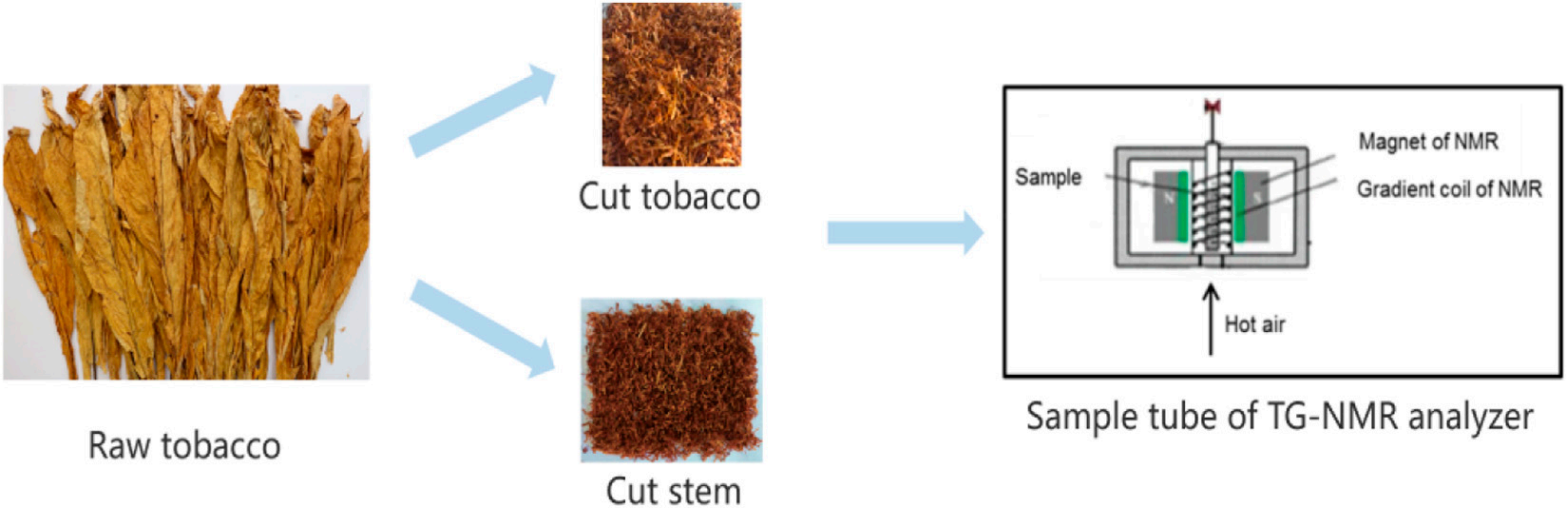
The preparation process for raw tobacco sample.

Two kinds of flue-cured tobacco biomass, including leaf and stem filaments, were used as the experimental materials, which were harvested in 2020 in Yunnan Province, China. As shown in Figure 1, raw tobacco were cut into tobacco strips with a width of 1mm, then placed in constant temperature and humidity environment to balance for 48 h.The initial moisture content of tobacco material was adjusted to 35% wet base moisture content. Then 2 g samples were put into the test tube of TG-NMR analyzer for drying analysis.

### 2.2 Instrument

**FIGURE 2:**
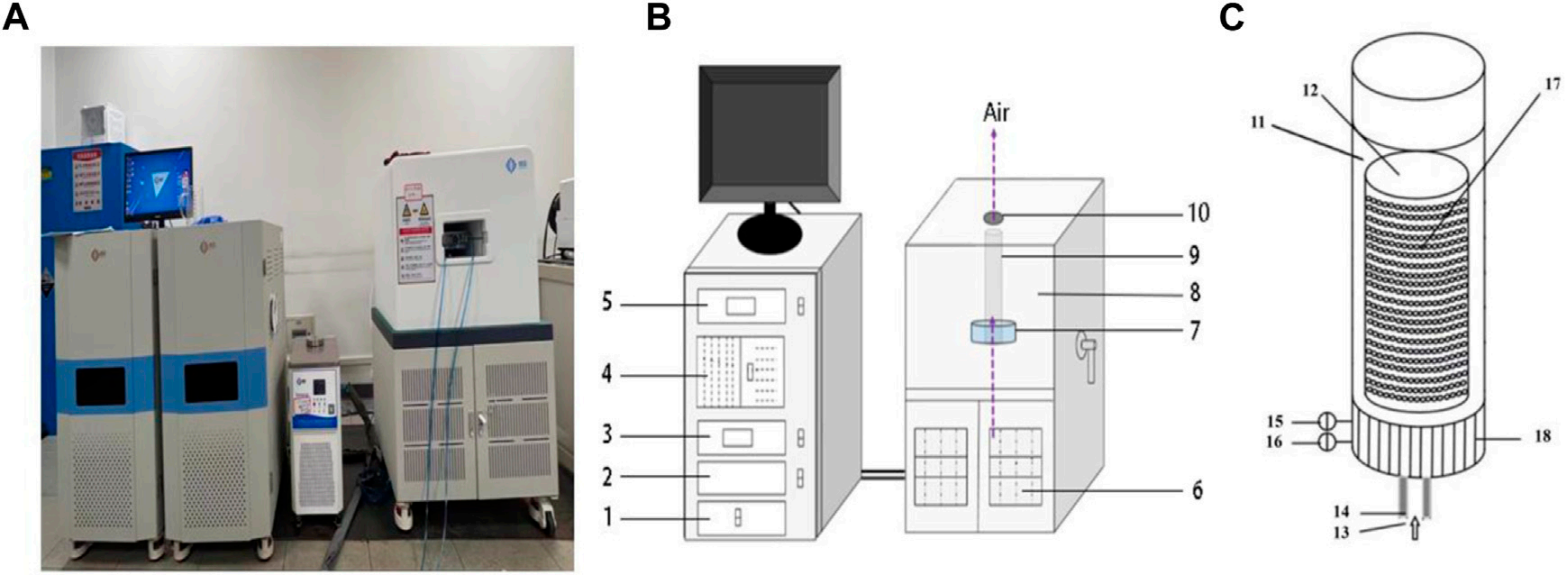
Physical map **(A)**, schematic diagram **(B)** and schematic diagram of the local structure of the sample chamber **(C)** of TG-NMR analyzer system.1-Gravimetry unit, 2-Radiofrequency unit, 3-Temperature controller unit, 4-Computer, 5-Moisturecontroller unit, 6-Heating equipment, 7-Coil, 8-Magnets cabinet, 9-Tube, 10-Sample position, 11-Probe barrel, 12-Sample chamber, 13-Airflow inlet,14-Insulation layer, 15-Airflow inlet temperature sensor, 16-Airflow inlet moisture sensor, 17-Opening, 18-Heat tracing assistance.

The TG-NMR analyzer consists of three parts, TG test part, temperature and humidity control part of drying air, as well as an situ low-field NMR test part (Suzhou Niumag analytical instruments Ltd., Suzhou, China). The physical map, schematic diagram and sample chamber of TG-NMR analyzer were shown in the Figure 2. The hot air can be adjusted within the temperature range of 50◦C–90°C and the humidity range of 0%–80%. The on-line NMR test section uses an NMR analyzer equipped with a 0.5 T permanent magnet for hydrogen proton relaxation measurements, which operates at 23 MHz.

After vacuum freeze drying, the pore structure of tobacco materials was analyzed using the mercury intrusion method with the Auto Pore IV 9500 mercury porosimeter (Micromeritics, United States), applying a maximum test pressure of 414 MPa. And by using helium, their density was detected by the Ultra PYC 1200e true density meter (Quantachrome, United States).

### 2.3 Methods

Tobacco materials of approximately 5 mm in length were manually selected and weighed into four portions of 2 g ± 0.001 g each for leaf and stem filaments. Hot air temperatures were set to 50, 60, 70, and 80°C, respectively, and tobacco samples were put in after the hot air temperatures were stabilized. During the test, the change in sample mass was obtained using a thermogravimetric analyzer to obtain the drying curve (moisture content vs. drying time). The moisture ratio (MR) was calculated as shown in the Eq 1 by assuming a drying hot air relative humidity of 0%:

$$MR=\frac{M-M_{eq}}{M_{0}-M_{eq}}$$ (1)

Where, M is the mass of the sample at any given time, Meq is the mass of the sample at equilibrium, M0 is the initial mass of the sample before drying commences.

In the study of tobacco drying kinetics, the diffusion of moisture is considered to be in accordance with Fick’s second law (Wang et al., 2020a). By assuming the one-dimensional diffusion of moisture in the tobacco material, Fick’s second law could be reduced simply as follows:

$$\frac{\partial M}{\partial t}=D_{e}\frac{\partial^{2}M}{\partial x^{2}}$$ (2)

Where M is the moisture content of the materials, %; x is the width of tobacco strips, m; t is the drying time, s; and De is the effective diffusion coefficient, m2/s.

A fillet width set to 1 mm, for an infinite slab with the spatial uniform moisture distribution and negligible external mass transfer resistance, the analytical solution is derived as Eq 3.

$$\frac{X}{X_{0}}=\frac{8}{\pi^{2}}\exp\left(-\frac{\pi^{2}Dt}{H^{2}}\right)+\frac{8}{9\pi^{2}}\exp\left(-\frac{9\pi^{2}D_{e}t}{H^{2}}\right)+\frac{8}{25\pi^{2}}\exp\left(-\frac{25\pi^{2}Dt}{H^{2}}\right)$$ (3)

Where, X is the moisture content of the slice at any given time, %; X0 is the moisture content of the slice before drying commences, %; De is the effective diffusion coefficient of moisture, m2. s−1; t is the drying time, s; n is the order.

The LF-NMR analyzer used in this study has a waiting time of 500 ms, an echo time of 250 µs, and 128 repetitions of sampling. At different drying time, RF signal was launched by the Carr-Purcell-Meiboom-Gill (CPMG) sequence, and received by NMR part of analyzer to obtain the T2 spectra of sample. In the present work, the NMR signal acquisition system was activated at 5, 10, 20, 30, 50, 70, 90 and 120 min to measure the spin-spin relaxation time signals of the tobacco in-process. The T2 spectra was obtained through numerical inversion using the model referred to in reference [23] (Guo et al., 2019). The model is shown in Eq. 4. The signal was normalized before inversion using the max-min normalization method.

$$I_{c_{P}MG}\left(t\right)=\sum_{i}I_{i}\exp\left(-\frac{t}{T_{2i}}\right)$$ (4)

## 3 Results

### 3.1 Water loss characteristics of tobacco in-process and T2 inversion spectrum

**FIGURE 3:**
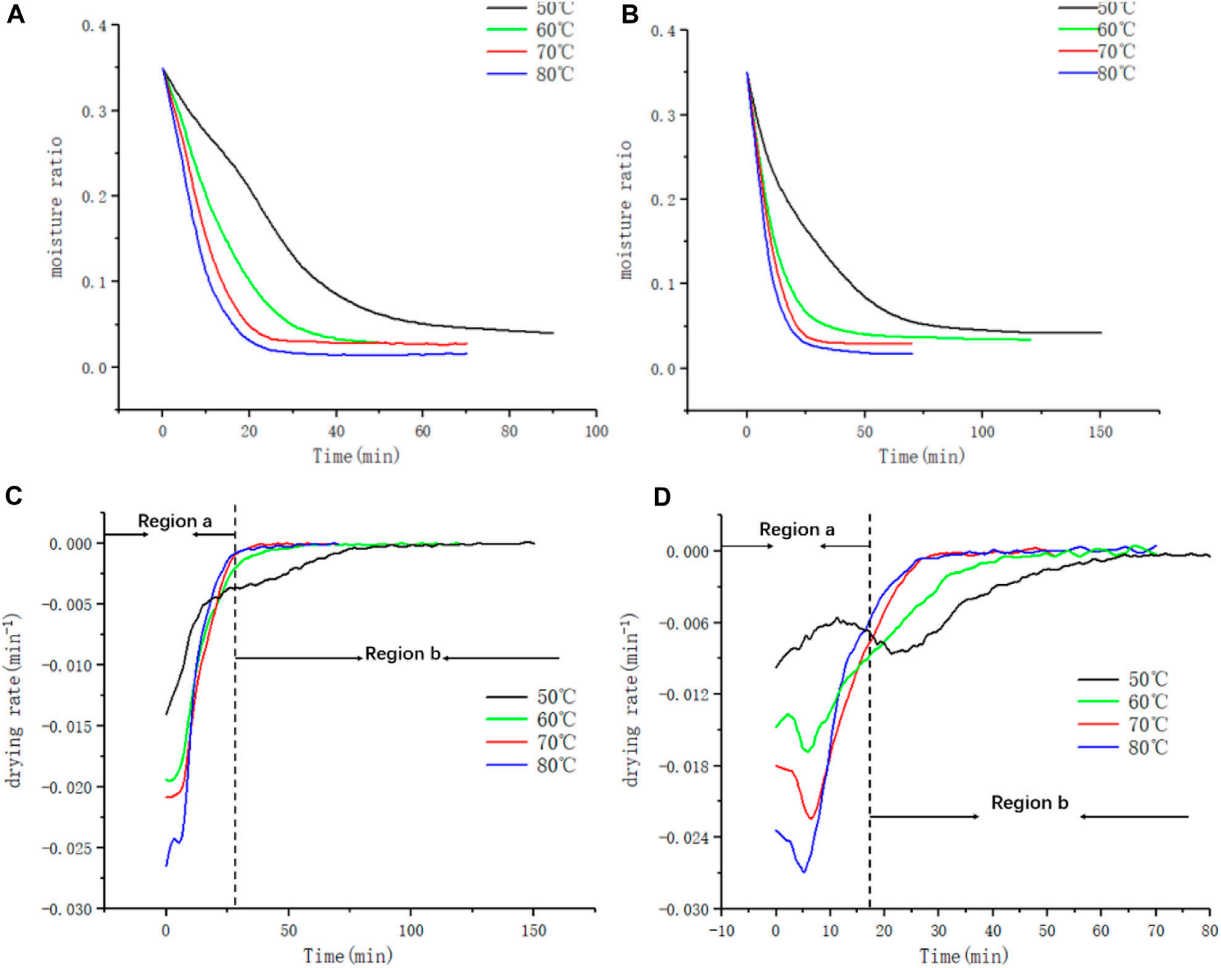
Weight loss curve and weight loss rate curve of **(A, B)** leaf filaments and **(C, D)** stem filaments.

Figure 3 shows the drying curves of leaf and stem waste under the drying conditions of 50, 60, 70◦C and 80°C. From the figure, it can be seen that the drying rate gradually decreases as the drying proceeds and the final moisture gradually tends to be constant.

The weight loss curve is differentiated to obtain the weight loss rate curve. If the drying process of tobacco materials could be regarded as a free water diffusion process, the dehydration rate should be proportional to the temperature of drying air according to the Clapeyron equation describing free water evaporation. Based on the characteristics of weight loss rate curves, the region a in Figures 3C, D is mainly corresponding to the loss stage of free water in the large pores of tobacco material. However, it was also found that the dehydration rate curves of leaf and stem filaments had the region b that was contrary to the above hypothesis, and the drying rate in this region was inversely proportional to temperature. Region b was related to the single-layer adsorbed water attached to the tobacco skeleton and free water in the small pores of tobacco material. Due to the earlier arrival of the drying endpoint at 70°C and 80°C, there was no significant difference in the drying rate at this temperature. The drying rate curves reflect the change in the different fugitive states of water during the drying of tobacco biomass.

In order to understand the water migration of tobacco biomass at different drying stages, the effective diffusion coefficients were calculated using the method based on Fick’s second law of diffusion. The effective diffusion coefficients of two tobacco materials calculated according to the above method are shown in Table 1. The coefficients of determination of both tobacco in-process in the fit are greater than 0.95, indicating that the effective diffusion model based on Fick’s second law can better reflect the drying kinetics of tobacco biomass. The effective diffusion coefficient of water in tobacco materials is within the range of 1.28 × 10-8∼6.72 × 10-8m2/s. The effective diffusion coefficient of leaf and stem filaments is related to the size of the pore volume and the structure. Compared to stem filaments, leaf filaments have larger pore volume and larger internal surface area, but more one-dimensional tubular structures were present in the stem filaments compared to the leaf filaments. At low temperatures, the thermal motion of water molecules is slow, and the structure plays a dominant role, resulting in a lower effective diffusion coefficient for stem filaments than for leaf filaments. At high temperatures, as the movement of water molecules accelerates, the size of the pore volume becomes the dominant factor, making the effective diffusion coefficient of leaf filaments greater than that of stem filaments.

**TABLE 1:** Correlation coefficient and effective diffusion coefficient of the tobacco biomass.

Hot air temperature/°C	Effective diffusion coefficient (m2/s)		R2	
	Leaf filaments	Stem filament	Leaf filaments	Stem filament
50	1.2814E-08	1.3131E-08	0.9830	0.9564
60	2.6457E-08	3.0866E-08	0.9677	0.9713
70	3.8648E-08	3.7870E-08	0.9717	0.9637
80	5.1876E-08	4.7726E-08	0.9764	0.9773

LF-NMR testing is achieved by aligning the magnetic moments of hydrogen atoms with an external magnetic field and generating dipole moments in hydrogen-containing fluids, recording the interaction of the spin of the hydrogen nucleus with the external dipole moment as the available signal. The peak in tensity and displacement in the T2 spectra obtained can be used to characterize the content and fugacity of hydrogen-containing fluids (e.g., water) in the sample. The low-field NMR T2 inversion spectra of tobacco materials usually show three to four peaks, which can be labeled as T21, T22, T23, T24from the shortest to longest relaxation times. The main peak T21 has the shortest relaxation time and the largest signal intensity, with a relaxation time in the range of 1 ms. According to the analysis of the T2 inversion spectra of roasted coffee beans (Mateus et al., 2007), the proton signal peaks with relaxation times around 100 ms were mainly caused by the hydrogen proton relaxation in the weakly polar oil molecules in the coffee material. By comparing the T2 inversion spectrum of dried tobacco with that of wet tobacco, It could be found that the main peak T21 in the proton signal peak after dehydration basically disappeared, while the intensity of other secondary peaks did not change significantly. It could be concluded that the main peak in the T2 inversion spectrum of tobacco material was the relaxation signal peak of hydrogen protons in water (Han et al., 2004). According to the multilayer adsorption theory (Pitombo and Lima, 2003b), the skeletal macromolecule hydrophilic groups will absorb water on the surface through hydrogen bonding, while hydrogen bonding will be formed again near the water layer because of polarization, generating a multilayer film of water molecules. According to the principle of low field NMR, the hydrogen proton relaxation time is shorter for bound water and monolayer water due to chemical bonding force or physical force, while the free water hydrogen proton relaxation time is longer. So the main peak relaxation time in T21 inversion spectrum can reflect the state of water fugacity of tobacco biomass.

**FIGURE 4:**
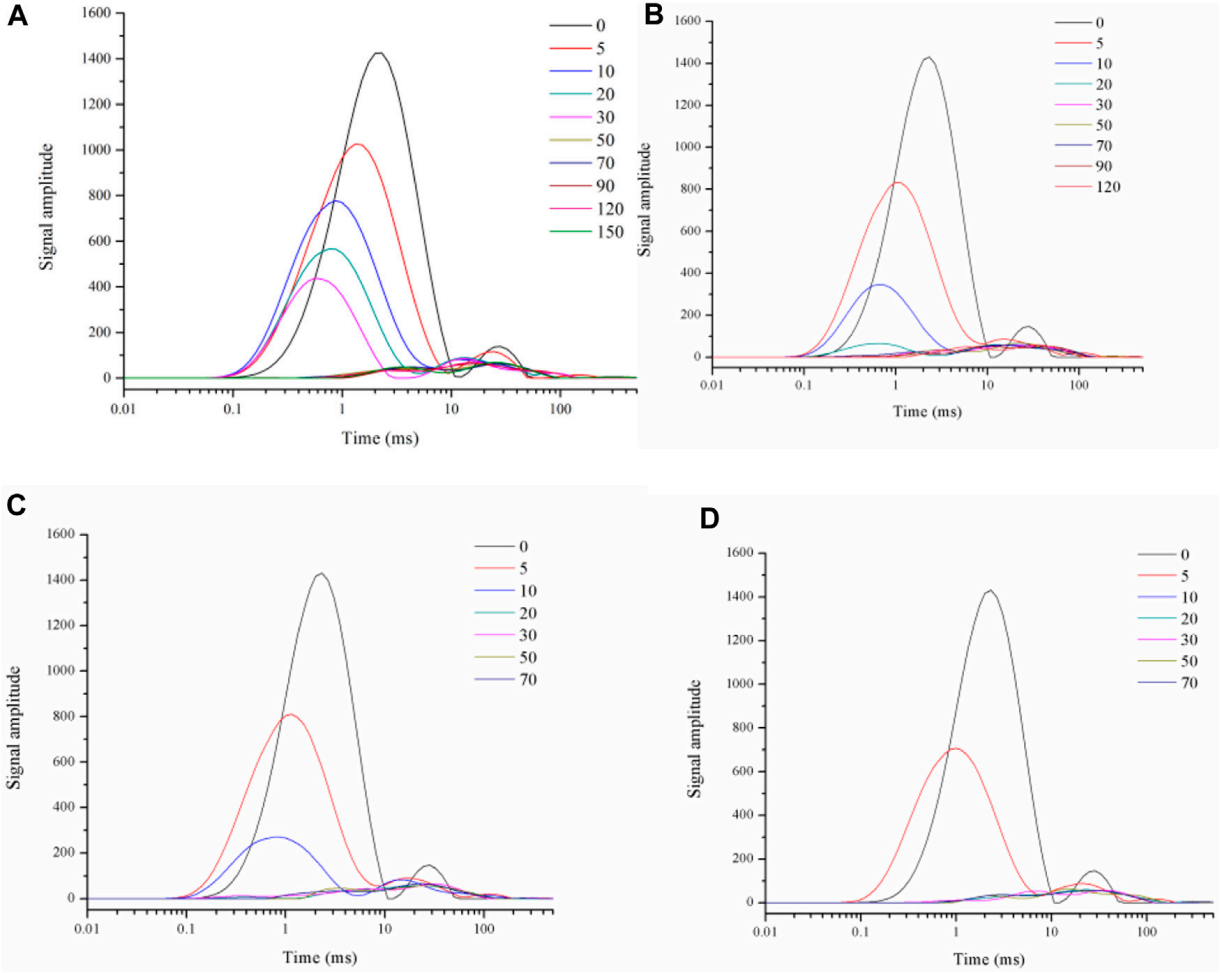
T2 spectrum of leaf filaments dried under the 50°C **(A)**, 60°C **(B)**, 70°C **(C)**, 80°C **(D)** hot air.

Figure 4 showed the inverse spectra of the spin-spin relaxation signal of roasted tobacco under different drying conditions. It can be seen that, comparing the T2 inverse spectra at different drying moments under a single drying temperature condition, the peak position of the T21 peak gradually shifts to the left as drying proceeds, and the intensity of peak gradually decrease. This indicated the loss of water and the decrease in water activity. At low moisture content, the monolayer moisture molecules in the material are directly attached to the tobacco skeleton, the hydrogen proton signal is weakened and the relaxation time is reduced. As the drying proceeded, the remaining water molecules are more firmly attached to the skeleton, and water loss becomes more difficult. In the meantime, the reducing magnitude of T21 peak relaxation time and peak signal magnitude becomes slower.

When the T2 inverse spectra for different hot air temperatures are compared, Figure 4 also showed the decrease of both the T21 peak relaxation time and signal intensity, as indicated that the drying intensity increased at the higher drying temperature. The moisture content at the late stage of drying was already below the acquisition limit of the instrument moisture signal, and the relaxation peaks of several drying moments appeared to overlap. At the end of drying, only the small peak of the oil substance was still always present and almost constant in size.

**FIGURE 5:**
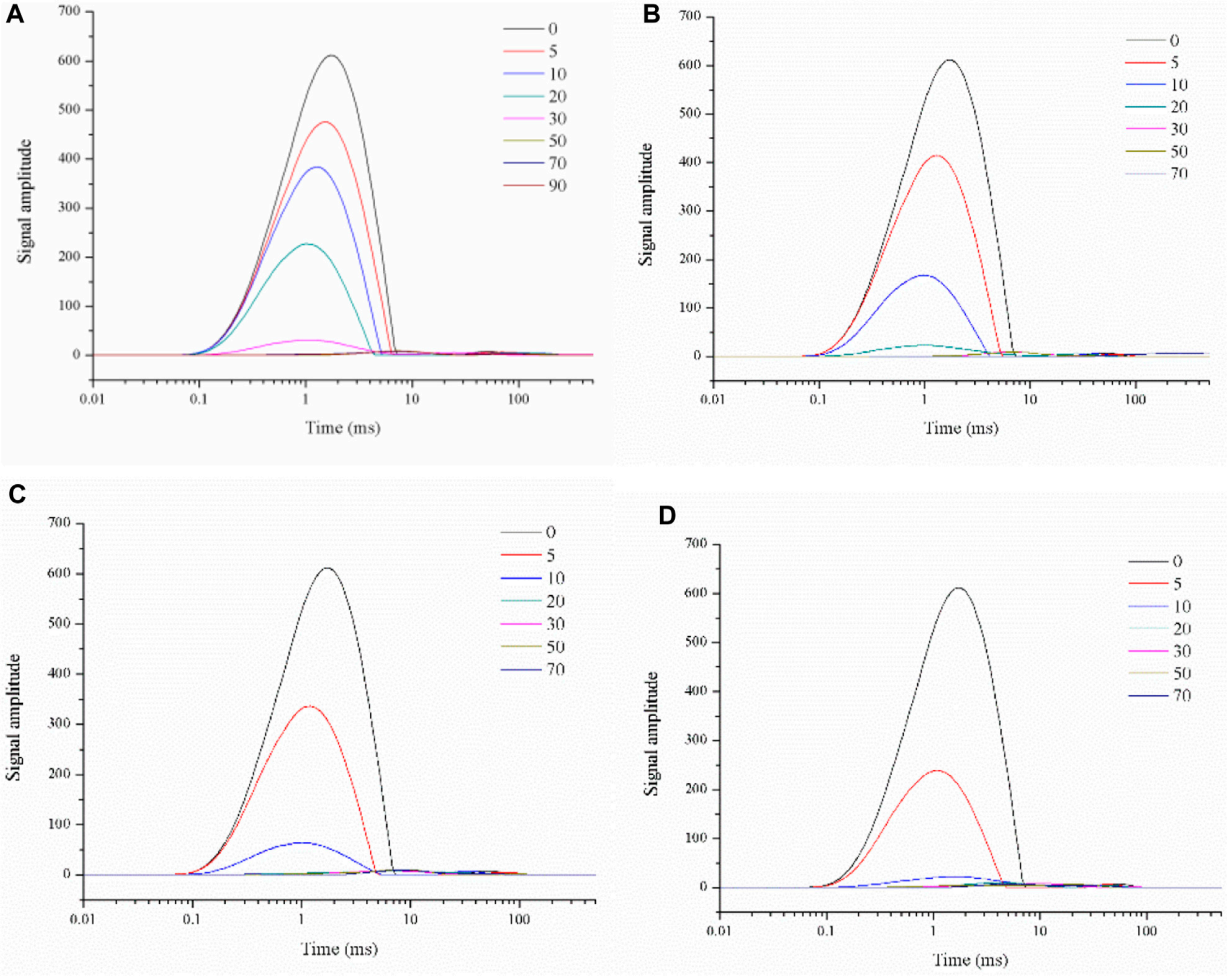
T2 spectrum of stem filaments dried under the of 50°C **(A)**, 60°C **(B)**, 70°C **(C)**, 80°C **(D)** hot air.

Figure 5 showed the inverse spectra of the spin-spin relaxation signals of stem filaments under different drying conditions. It can be seen that the results for different drying intensities and different drying moments are similar to those for leaf filaments waste, with the T21 peak movement to the left and the peak intensity gradually decreasing as the drying progresses. However, the T21 peak position shifted less during the drying process for the stem compared to the T2 inverse spectrum for the leaves.

### 3.2 Association between changes in pore structure and water fugacity of tobacco in-process

Tobacco materials are porous materials, and the microscopic pore structure affects the moisture migration and its residence state. As a deformable plant-based porous medium, tobacco has obvious shrinkage deformation phenomenon during the drying process, and its internal volume and microstructure have large changes during the drying process (Wang et al., 2020b). The change of pore size and distribution will affect the moisture residence state and migration process. In order to analyze the change of pore volume of tobacco biomass during drying, the unit pore volume V is defined as Eq 5.

$$V=\frac{1}{\rho a}-\frac{1}{\rho t}$$ (5)

ρ a - apparent density of leaf filaments, g/cm3; ρ t - true density of leaf filaments, g/cm3.

Taking the drying temperature of 80°C as an example, the results of the apparent density and true density tests of the leaf and stem filaments are shown in the Table 2.

**TABLE 2:** True and apparent densities of two kinds of tobacco biomass at different moisture contents.

Moisture content/%	Leaf filaments		Stem filaments	
	Apparent density g/cm3	True density g/cm3	ApparentDensity g/cm3	True density g/cm3
30	1.0366	1.6563	1.1246	1.6542	
25	1.0535	1.4250	1.1397	1.6316	
20	1.0685	1.4000	1.1432	1.5689	
15	1.0678	1.3647	1.1541	1.5122	
10	1.0989	1.2279	1.1356	1.3867	

**FIGURE 6:**
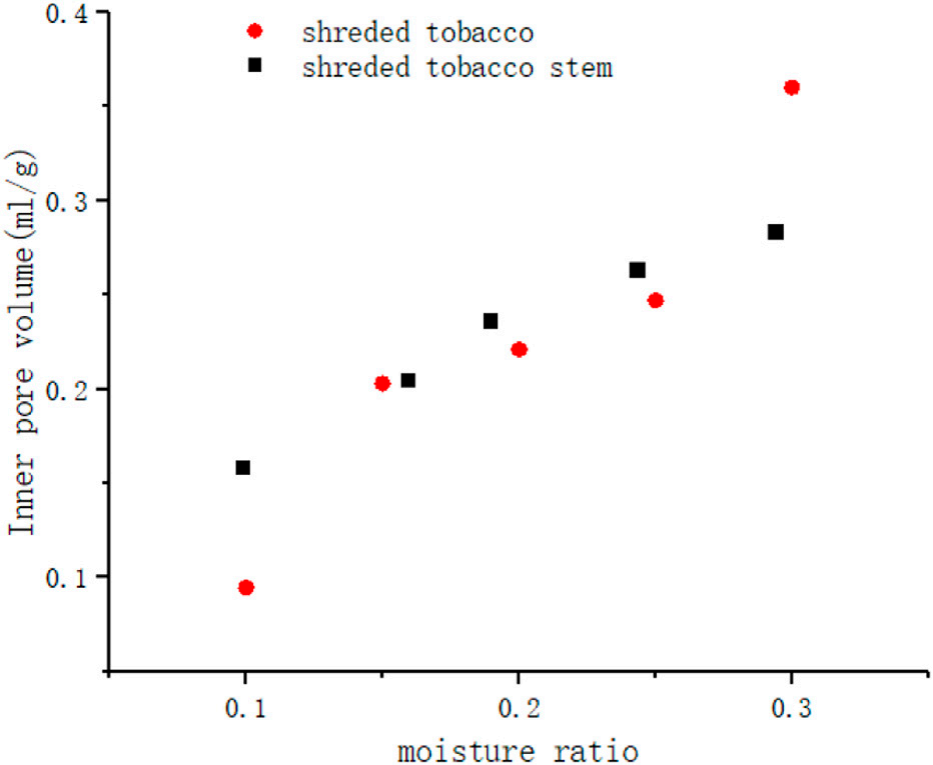
Inner pore volume of two kinds of tobacco biomass under different moisture.

From the Table 2, the variation law of the inner pore volume of leaf and stem waste at different moisture contents during the drying process could be obtained according to the equation, as shown in Figure 6.

As can be seen, the pore volume decreases by nearly 75% for leaf filaments and 60% for stem filaments when the moisture content of tobacco biomass decreased from 30% to 10%. According to the SEM analysis in previous work (Han et al., 2004), the inner pores of leaf filaments were composed of intracellular pores and intercellular pores, and these pores were of different sizes and interwoven to form a complex meshwork structure composed of cytoskeleton. However, more one-dimensional tubular structures were present in the stem filaments compared to the leaf filaments. The two tobacco materials have different microstructures and pore skeletons. During the drying process, the water in the internal pores of plant tissue evaporates to form a curved liquid surface, which will cause shrinkage stress on the pore wall. The pore structure formed by the lamellar skeleton of the leaf filaments is more compressible under shrinkage stress and its internal pore volume is reduced more, while the higher deformation stress of the one-dimensional tubular structure of the stem filaments hinders the reduction of the internal pore volume. The reduction in pore volume in tobacco materials may lead to an increase in the number of small pores with lower activity endowed in them, which is partly responsible for the decrease of the relaxation time of the T21 peak. Moreover, according to the multilayer adsorption theory, the free water loss leads to a tight connection of the remaining water to the tobacco skeleton, also then cause a reduction in water activity.

**FIGURE 7:**
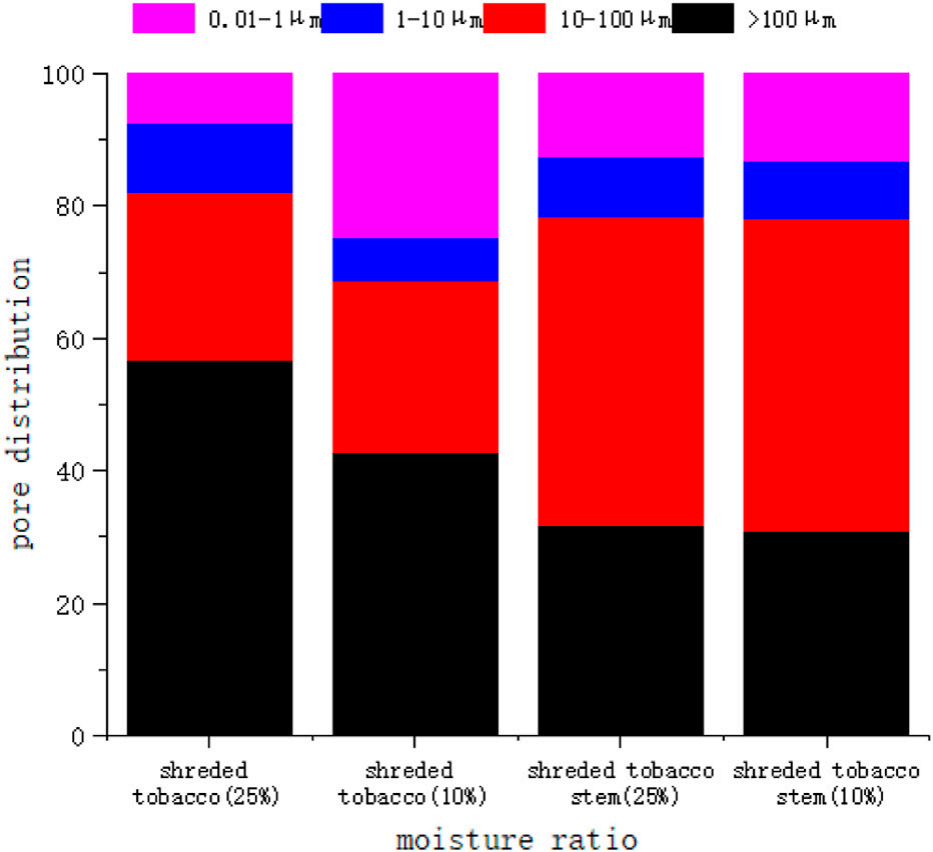
Pore distribution of leaf and stem filaments at 25% and 10% moisture content.

To further explain the differences in T21 peak relaxation time changes during the drying process of two tobacco materials, this study characterized the pore distribution using the mercury-pressure method for leaf and stem filaments with 25% and 10% moisture content. The results are shown in Figure 7 below.

The above figure shows that the dry shrinkage in leaf filaments significantly changing pore distribution. At 25% moisture content, pores larger than 100 µm make up over half of the total. Here, most leaf filaments are macropores, with fewer mesopores and micropores. In these macropores, most of the water is free and highly active, leading to longer T21 peak relaxation times. At 10% moisture content, 1–100 µm pores shrink, and 0.01–1 µm pores increase substantially. Tobacco’s pore distribution has shifted towards smaller pores. Water in these smaller pores binds to the tobacco skeleton *via* forces like capillary action. This water has higher adsorption energy but lower activity than the water in larger pores. Consequently, leaf filaments experience a more significant decrease in T21 peak relaxation time. In contrast, for stem filaments, the pore distribution was not significantly altered with 25% and 10% moisture content, although the pore volume was also reduced. This shows that the decrease in T21 peak relaxation time in leaf filaments is mainly due to reduced water activity. This reduction is caused by the evaporation of free water, which thins the water layer adsorbed on the tobacco skeleton. The adsorption forces such as capillary forces are stronger than the tobacco skeleton’s forces on water. This means water activity weakens less in stem filaments compared to leaf filaments. As a result, the decrease of the T21 peak’s relaxation time is more pronounced in leaf filaments than in stem filaments.

### 3.3 Spatial migration pattern of water during drying of tobacco in-process

Based on the patterns derived from LF-NMR data of leaf and stem filaments, combined with the pore changes during drying, hypotheses are given for the water migration behavior of tobacco in-process at region a and region b. In region a, located at the early stage of drying, labile free water loss occurs for both leaf and stem filaments. During this process, water molecules mainly overcome intermolecular hydrogen bonds. Water is lost more easily in large pores, reducing resistance to mass transfer. Thus, in region a, the drying rate is proportional to temperature. The higher the temperature, the greater the drying rate. As drying continues, most of the easily lost free water completely evaporates. The more difficult to evaporate water remains in the tobacco, including monolayer water on the skeleton and water in the micropores. In region a, the drying rate is low at low temperatures. As a result, not all susceptible free water is lost in this stage. Therefore, in region b, the drying rate becomes inversely proportional to temperature.

**FIGURE 8:**
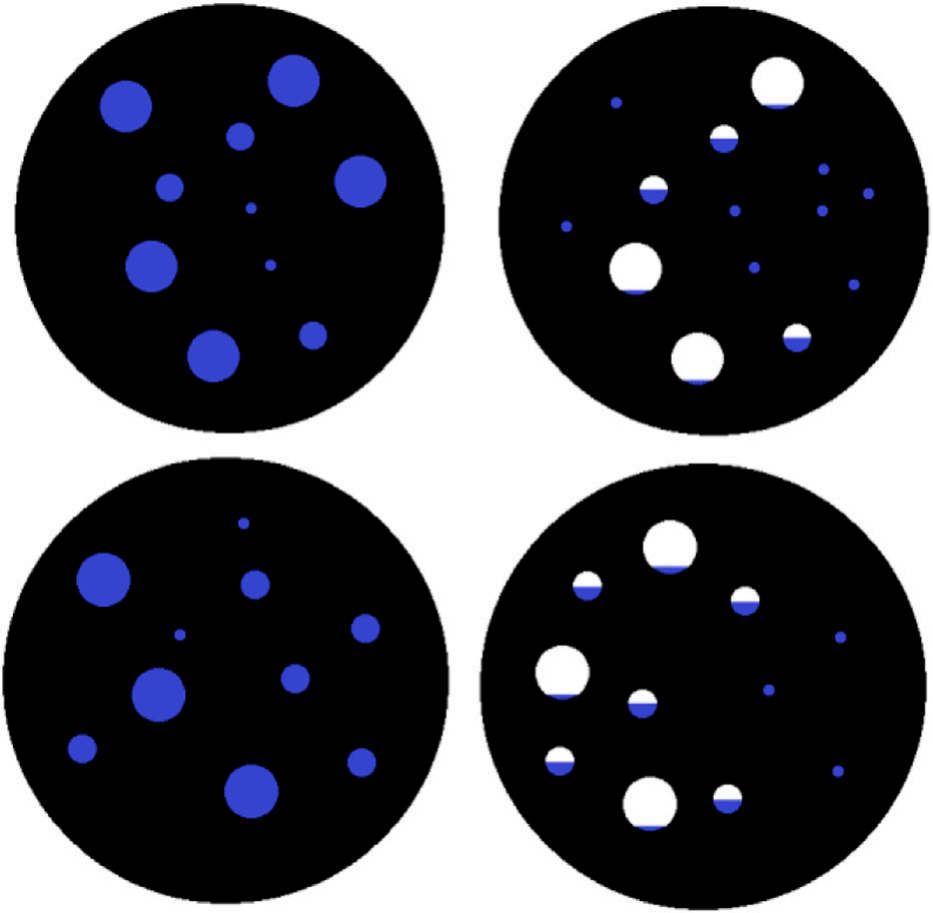
Moisture spatial migration law.

Based on the pattern above, Figure 8 shows the different water spatial migration behaviors of tobacco leaf and stem waste. In drying, the tobacco preferentially loses free water in the large pores, and the activity of water weakens as the pores shift towards smaller sizes and increase in number. Stem filaments also preferentially lose free water in the larger pores in drying. Since the pore distribution of stem has little change with decreasing moisture, the graph indicates that the original large pores become medium pores, and the medium pores develop into small pores.

### 3.4 Relationship between adsorption energy and moisture loss of leaf and stem filaments

Water absorbed by the micropores and tobacco structure decreases water activity, subsequently impacting the relaxation time of the hydrogen proton low-field NMR T2 inversion spectrum. The moisture adsorption capacity of tobacco material can be expressed by the adsorption energy, where higher adsorption energy implies lower activity of moisture.

Drying can be characterized as a typical three-stage mass transfer process. First, liquid-phase molecules move from the condensed phase to the surface of liquid-phase water, which is a re-versible process in terms of chemical kinetics. Second, water molecules vaporize and diffuse from the surface layer into the gas phase. Finally, water molecules diffuse from the gas phase within the material to the material’s surface. Due to the influence of convective mass transfer during hot air drying, the diffusion rate of water molecules from the pore structure to the surface of the tobacco material is accelerated, and the process can be considered uncontrolled. According to the theory of Donskoy (Donskoy et al., 2019), the adsorption energy equation can be represented as Eq 6.

$$g_{l}^{ad}=-RTln\left(\frac{J_{ev}}{J_{ev}^{w}}\right)$$ (6)

$g_{l}^{ad}$ is the free energy of moisture adsorption, R is the gas constant, T is the temperature, and $J_{ev}$ is the diffusion flux, and $J_{ev}^{w}$ is the initial diffusion flux.

Donskoy’s theory serves as the foundation for understanding moisture fugacity. It relies on reasonable approximations to calculate moisture adsorption energy during drying, which is based on initial drying rate and drying rate data.

**FIGURE 9:**
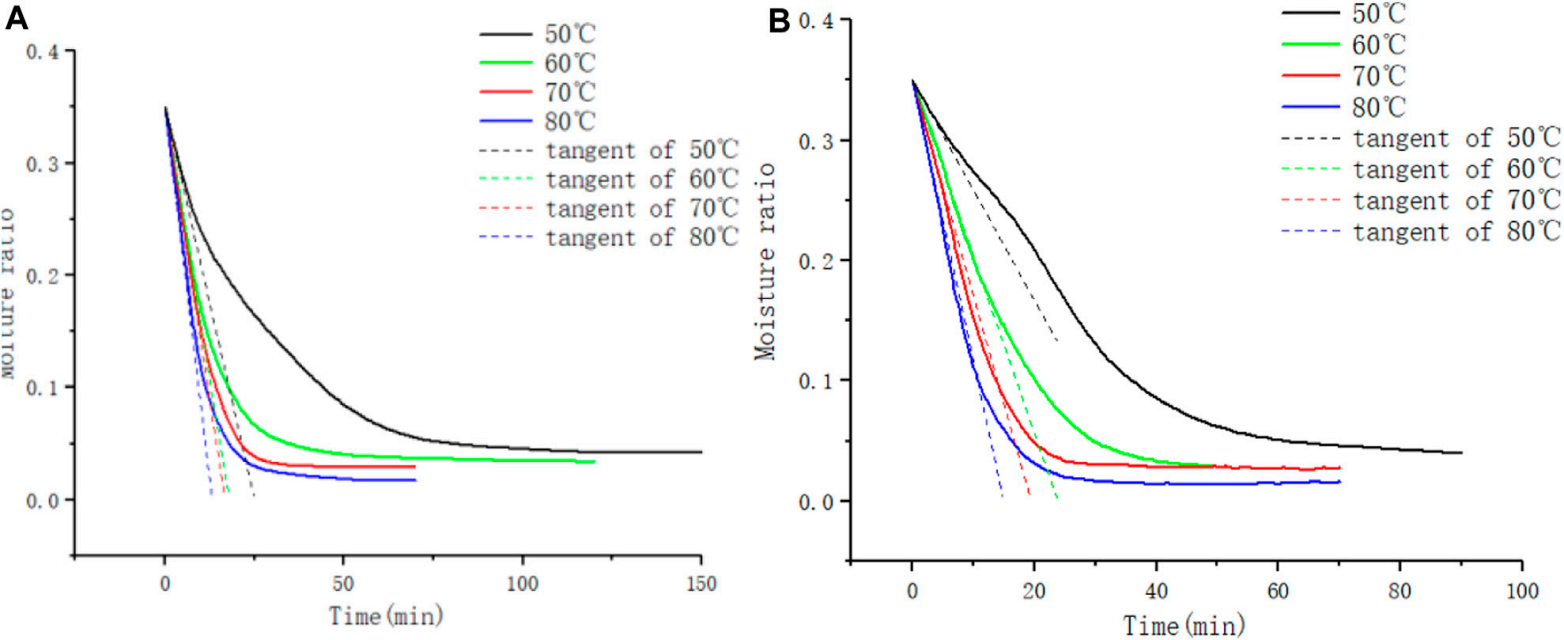
**(A)** $J_{ev}^{W}$ of tobacco leaf filaments, **(B)** $J_{ev}^{W}$ of tobacco stem filaments.

A tangent is drawn on the thermogravimetric curve at the point where the drying process starts, precisely when the water content reaches 0.35. The slope of this tangent, as shown in Figure 9, represents the initial drying rate. Following the description above, this value is converted to obtain the initial diffusion flux $J_{ev}^{W}$ . For leaf filaments, the tangent slopes on the thermogravimetric curve were −0.01946 at 60°C, −0.02084 at 70°C, and −0.02659 at 80°C, while for stem filaments, they were −0.0147 at 60°C, −0.018 at 70°C, and −0.02342 at 80°C. The initial diffusive flux for both types of tobacco in-process was higher for leaf filaments compared to stem filaments. This difference can be attributed to the wider distribution of macropores in leaf filaments, making it easier to initiate the drying process for water within them. The adsorption energy data for various moisture levels in leaf and stem filaments were obtained using Donskoy’s equation, in combination with drying rate data at different temperatures, as illustrated in Figure 9 below.

The sorption energy-moisture curve varies for each material, depending on the drying temperature and the material properties. Moisture sorption energy is close to zero at high water content, when the water layer is thicker and more active according to multilayer sorption theory. At around 20% water content, the adsorption energy starts to increase rapidly, which means that the water layer with high adsorption energy starts to evaporate, and it can be assumed that the tobacco skeleton is chemically or physically bound to the water at this time, which represents that water may need to overcome capillary or weak chemical bonding forces to pass from the liquid phase to the gas phase. The main components of the tobacco in-process are cellulose, lignin, and hemicellulose, and the calculations of moisture sorption energy in this study are roughly in the same order of magnitude as published data on cellulose (Nikitin, 2023). In agreement with our expectations, the adsorption energy of leaf filaments was greater than that of stem filaments at the three temperatures, which can explain the greater decrease in T21 peak relaxation time of leaf filaments compared to that of stem filaments when combined with the data on leaf and stem pore.

## 4 Conclusion

In this study, TG and LF-NMR techniques were employed to explore the moisture fugitive migration behavior of two types of tobacco during the drying process. LF-NMR data were collected for various moisture contents in leaf and stem filaments, revealing that as the drying process progresses, the relaxation time of the T21 peak gradually decreases, and the peak’s signal intensity also gradually reduces. Additionally, the T21 peak relaxation time exhibited a more pronounced decrease in leaf filaments compared to stem filaments. This was due to a larger reduction in pore volume and a significant shift toward smaller pores that bind water with higher adsorption energy, leading to a greater reduction in water activity. Furthermore, the spatial migration characteristics of water during the drying of leaf and stem filaments were proposed. The results of moisture adsorption energy calculations indicated the adsorption energy of leaf filaments was greater than that of stem filaments. This is because the changes in pore distribution and the degree of pore volume reduction are more significant in leaf filaments during the drying process. This study provide fundamental theoretical references for improving the tobacco composting process and refining the moisture migration mechanism in tobacco processing.

## Data Availability

The original contributions presented in the study are included in the article/Supplementary material, further inquiries can be directed to the corresponding authors.
